# IMPACT OF A SOCIAL CONNECTION PROGRAM ON OLDER ADULTS DURING THE COVID-19 PANDEMIC

**DOI:** 10.1093/geroni/igac059.2615

**Published:** 2022-12-20

**Authors:** Rachel Ungar, Rifky Tkatch, Yan Cheng, Sandra Kraemer, Michael McGinn, Ellen Wicker

**Affiliations:** Optum Labs, Oak Park, Michigan, United States; Optum Labs, Minnetonka, Minnesota, United States; Optum Labs, Minnetonka, Minnesota, United States; UnitedHealthcare, Minnetonka, Minnesota, United States; Optum Labs, Minnetonka, Minnesota, United States; ASI, Inc., Washington, District of Columbia, United States

## Abstract

**Background:**

Research demonstrates social connections decrease loneliness and improves life satisfaction among older adults. However, the COVID-19 pandemic limited social connectedness, specifically for older adults. Thus, programs aimed to increase social connectedness among older adults are integral to their well-being. Purpose: The primary objective of this study was to determine if the telephonic Peer-to-Peer (P2P) program could improve social connectedness and reduce loneliness among older adults. A secondary objective was to improve life satisfaction and social support.

**Methods:**

Eligible older adults (age 65+) were recruited via outbound calls and/or a mailer. Participants were mailed a T1 survey, completed intervention training, and matched into a dyad. The matched dyad engaged in weekly telephone calls for 12 weeks. Post 12 weeks, participants completed a T2 survey, and a T3 four weeks later.

**Results:**

A total of 475 participants completed a T1, and 125 dyads (250 individuals) completed a T3. Older females were more likely to participate (77% female, 44% 75+ old). Individuals who were lonelier at baseline showed a significant improvement in loneliness throughout the program, as well as improvement in social support, life satisfaction, and subjective happiness.

**Conclusion:**

Results from this program showed significant improvement in psychosocial well-being outcomes for lonely older adults. This program took place during the initial months of the COVID-19 pandemic and demonstrated success for lonely older adults with limited technology who may have been socially isolated during this time.

